# Coupling freedom from disease principles and early warning from wastewater surveillance to improve health security

**DOI:** 10.1093/pnasnexus/pgac001

**Published:** 2022-03-02

**Authors:** David A Larsen, Mary B Collins, Qian Du, Dustin Hill, Tabassum Z Insaf, Pruthvi Kilaru, Brittany L Kmush, Frank Middleton, Abigail Stamm, Maxwell L Wilder, Teng Zeng, Hyatt Green

**Affiliations:** Department of Public Health, Syracuse University, Syracuse, NY 13244, USA; Department of Environmental Studies, SUNY ESF, Syracuse, NY 13210, USA; Quadrant Biosciences, Inc, Syracuse, NY 13210, USA; Department of Environmental Studies, SUNY ESF, Syracuse, NY 13210, USA; Bureau of Environmental and Occupational Epidemiology, New York State Department of Health, Albany, NY 12237, USA; Department of Epidemiology and Biostatistics, University at Albany, Albany, NY 12222, USA; Department of Public Health, Syracuse University, Syracuse, NY 13244, USA; Department of Public Health, Syracuse University, Syracuse, NY 13244, USA; Department of Biochemistry and Molecular Biology, SUNY Upstate Medical University, Syracuse, NY 13210, USA; Bureau of Environmental and Occupational Epidemiology, New York State Department of Health, Albany, NY 12237, USA; Department of Environmental Biology, SUNY ESF, Syracuse, NY 13210, USA; Department of Civil and Environmental Engineering, Syracuse University, Syracuse, NY 13244, USA; Department of Environmental Biology, SUNY ESF, Syracuse, NY 13210, USA

**Keywords:** health security, wastewater surveillance, freedom from disease, SARS-CoV-2

## Abstract

Infectious disease surveillance is vitally important to maintaining health security, but these efforts are challenged by the pace at which new pathogens emerge. Wastewater surveillance can rapidly obtain population-level estimates of disease transmission, and we leverage freedom from disease principles to make use of nondetection of SARS-CoV-2 in wastewater to estimate the probability that a community is free from SARS-CoV-2 transmission. From wastewater surveillance of 24 treatment plants across upstate New York from May through December of 2020, trends in the intensity of SARS-CoV-2 in wastewater correlate with trends in COVID-19 incidence and test positivity (*⍴* > 0.5), with the greatest correlation observed for active cases and a 3-day lead time between wastewater sample date and clinical test date. No COVID-19 cases were reported 35% of the time the week of a nondetection of SARS-CoV-2 in wastewater. Compared to the United States Centers for Disease Control and Prevention levels of transmission risk, transmission risk was low (no community spared) 50% of the time following nondetection, and transmission risk was moderate or lower (low community spread) 92% of the time following nondetection. Wastewater surveillance can demonstrate the geographic extent of the transmission of emerging pathogens, confirming that transmission risk is either absent or low and alerting of an increase in transmission. If a statewide wastewater surveillance platform had been in place prior to the onset of the COVID-19 pandemic, policymakers would have been able to complement the representative nature of wastewater samples to individual testing, likely resulting in more precise public health interventions and policies.

Significance Statement Wastewater provides a representative sample and can be easily collected and analyzed to understand the health of the community. Numerous infectious disease pathogens can be found in wastewater, including SARS-CoV-2. Using SARS-CoV-2 as a case study, we show that wastewater surveillance can improve health security by confirming freedom from disease transmission when a pathogen is absent from a community and provide an early warning for emerging pathogens. We apply these principles to SARS-CoV-2, and hypothesize that they will be useful for many other infectious disease pathogens including but not limited to HIV, tuberculosis, dengue, zika, and malaria.

## Introduction

Infectious disease surveillance, or monitoring trends in the transmission of communicable diseases, is an important pillar of public health and is fundamental to the health security of a population (1–3). Infectious disease surveillance systems are typically event-based, designed to measure abnormal increases in either identified cases of a laboratory-diagnosed pathogen or identified cases, where individuals display a set of signs and symptoms (syndromic surveillance) (4). Although infectious disease surveillance is necessary to identify and control disease outbreaks (5), novel pathogens are incredibly difficult to incorporate into existing event-based surveillance networks. For example, by the time that sudden acute respiratory coronavirus 2 (SARS-CoV-2) was first noticed via an increase of unexplainable cases of pneumonia (6), the pathogen had spread widely through the community in Wuhan, China. Subsequently an event-based surveillance approach was not a readily feasible strategy to confirm that connected communities were free from SARS-CoV-2 transmission as the pathogen spread across the globe. Despite heroic efforts to scale COVID-19 diagnostics as quickly as possible, the virus spread more rapidly, exceeding testing capacity early in the pandemic. Without an operational event-based surveillance strategy based upon virus diagnostics, officials in the United States and elsewhere had to rely on syndromic surveillance as SARS-CoV-2 spread—basing public health interventions on increases in hospitalizations and deaths. Since timely, geographically granular information regarding transmission was not available, public health officials were left with few management options, other than to call for a halt to nonessential businesses and gatherings. Equipped with timely and geographically granular information regarding the transmission of an emerging pathogen, public health officials would have more resource flexibility and intervention tools to potentially intervene earlier in an outbreak before it becomes widespread in the community. Importantly, public health officials need to know where transmission is not occurring (7), or at least controlled.

Wastewater surveillance addresses challenges and limitations of event-based surveillance systems by assessing infectious disease transmission potential within the entire population served by a sewage system. A single sample conveys information independent of individuals’ symptoms, health-seeking behavior, or the health system's availability of diagnostic testing (8). The genetic material from SARS-CoV-2 (as with many infectious pathogens) is shed in human feces and urine (9–11). Finding that genetic material in wastewater can serve as an early indication of a pathogen in a community, as demonstrated in the Netherlands, Paris, Connecticut, and elsewhere (12–15). Perhaps more importantly, as we show herein, the absence of genetic material in the wastewater can give confidence that a community is free from transmission of that pathogen. Tracking infectious disease transmission through wastewater is not a novel approach—it goes back more than 100 years (16–18), and saw broadscale application in the later 1900s to identify outbreaks of polio (19). Wastewater surveillance in some cases even allowed for vaccine campaigns to control community polio outbreaks before any polio-caused paralysis occurred (20, 21). From May through December of 2020, we analyzed 1,528 wastewater samples from 76 sewersheds nested within 24 wastewater treatment plants in 14 counties throughout upstate New York (Figure S1, Supplementary Material) using previously validated methods (22). Although not repeated here, we previously demonstrated that these methods are sensitive enough to detect < 10 reported daily cases per 100,000 residents in most areas and that normalizing SARS-CoV-2 concentrations to levels of crAssphage strengthens associations between wastewater SARS-CoV-2 concentrations and infection rates (22). Here, we compare levels of SARS-CoV-2 RNA found in the wastewater to measures of SARS-CoV-2 transmission as reported by the New York State Department of Health. Using freedom from disease principles (23) benchmarked to transmission intensity guidelines from the US Centers for Disease Control and Prevention (CDC) (24), we then assessed the ability of wastewater surveillance to confirm that SARS-CoV-2 transmission was either under control or absent. By comparing spikes in SARS-CoV-2 as measured in wastewater to spikes in event-based surveillance measures of SARS-CoV-2 transmission, we determined that wastewater surveillance provided early warning of localized outbreaks in 3 communities. Together, these analyses provide compelling evidence that a wastewater surveillance network would greatly increase health security.

## Materials and Methods

### Wastewater testing

Participating wastewater treatment plants, or Arcadis during the New York State pilot, sent a 24-hour composite of 250mL untreated wastewater to the Quadrant Biosciences wastewater analysis laboratory either weekly, twice weekly, or three times weekly depending upon services contracted. Quadrant Biosciences purified and quantified SARS-CoV-2 viral nucleic acid levels contained in wastewater samples by using the ultracentrifugation through sucrose cushion (UltraSucrose) technique followed by qRT-PCR as detailed elsewhere (22). Briefly, wastewater was added to a centrifuge tube before adding sucrose solution under the wastewater creating two distinct layers. After ultracentrifugation, the supernatant was removed and the pellet containing nucleic acids was resuspended. Total nucleic acids were extracted from eluted pellets and used immediately as the template for quantification of SARS-CoV-2 and crAssphage DNA and crAssphage RNA. To aid interpretation, SARS-CoV-2 wastewater RNA levels were classified into three distinct categories prior to data analysis. qPCR was run in triplicate. Samples that had all three qPCR replicates amplify above the limit of quantification (LOQ) of 5 genome copies per reaction were classified as quantifiable. Because both assays were able to amplify 5 copies per reaction consistently, samples that had at least one qPCR replicate amplify with a cycle-time threshold (Ct) < 40 were considered detected but not quantifiable (DNQ). Many of the samples classified as DNQ had one or two qPCR replicates above the LOQ of 5 copies but were still conservatively classified as DNQ for our analysis. Samples that had no amplification in any of the three wells (i.e., all three wells were “undetermined”, Ct > 45) were considered below the limits of detection (BLOD), i.e. a “negative” or non-detected sample. The following equation was used to normalize SARS-CoV-2 quantities to the level of fecal material in each sample as indicated by crAssphage DNA concentrations: log10(SARS-CoV-2):log10(crAssphage DNA). We imputed a level of 3.5 copies for DNQ samples and 1 copy for BLOD samples prior to log-transformation.

### COVID-19 case data

COVID-19 case data was pulled from the Electronic clinical Laboratory Reporting System (ECLRS). Every licensed professional authorized by the Department of Health Physician Office Laboratory Evaluation Program to administer a test for COVID-19 or influenza is required to report such results immediately (not more than 3 hours) to the Department of Health through ECLRS when a result is received (25, 26). COVID-19 cases and tests were retrieved from the New York State ECLRS, addresses were matched with tax parcel data to determine whether the household was connected to public sewer, and then geocoded to sewershed geographies before aggregation into daily numbers. Test positivity was defined as the number of COVID-19 cases divided by the number of COVID-19 tests conducted. The number of active cases was estimated as each COVID-19 case lasting 10 days from diagnosis. We utilized seven-day averages for test positivity and incidence, but not for active cases. We estimated simple Pearson correlations between levels of SARS-CoV-2 RNA in wastewater and measures of incidence, active cases, and test positivity.

### Classification of COVID-19 transmission

We used the CDC’s guidelines for classifying transmission into low, moderate, substantial, or high depending on the number of weekly cases of COVID-19 per 100,000 population as well as test positivity (24). From our results we showed a three-day lead time between wastewater levels and active cases, and so we positioned our week of COVID-19 cases and test positivity accordingly. If incidence and test positivity fall into different risk categories, the CDC recommends taking whichever is higher. For all analyses herein, incidence was higher than test positivity.

### Sewershed population estimates

We calculated estimates for the 2020 population within each sewershed using R statistical software version 4.0.0 (27). We first estimated the 2010 population for each sewershed using an overlay of 2010 US census blocks on top of the sewershed boundaries. We calculated the proportion of the area for partial block overlap and then assigned a proportional 2010 decennial population of the block to the sewershed assuming equal distribution of the population in the blocks. We then aggregated the apportioned values to get a total population estimate for the sewershed. We repeated this procedure using 2010 decennial population data for the block group and 2018 American Community Survey (ACS) data for the block group to get 2010 and 2018 population by sewershed based on block groups. We used these values to estimate the rate of population change per sewershed using equation 1. We then applied this average annual change to the sewershed population based on the block data from 2010 and estimated the population after ten years of growth using equation 2 to calculate 2020 population estimates. The “tidycensus” R package provided population estimates (28) and the “tigris” package provided geometry data (29).

(1) \begin{eqnarray*} {Annual\, growth\, rate} =\left(\frac{2018_{pop}-2010_{pop}}{2010_{pop}}\right)/8\, {years} \end{eqnarray*}

(2) \begin{eqnarray*} 2020\, {Population\, estimate} = 2020_{pop} \times (1+ {annual\, growth\, rate}^{10} \end{eqnarray*}

### Freedom from transmission estimates

In order to estimate the limits of detection of wastewater surveillance we first categorized wastewater results into not detected, detected but below the limits of quantification, and quantifiable. We then examined the reported cases and test positivity in the sewershed as a function of each of these categories. We calculated sensitivity to confirm absence of transmission as among all wastewater tests where SARS-CoV-2 RNA was not detected, the proportion of sewersheds reporting 0 daily incident COVID-19 cases at the time of the wastewater sample. Following the calculated limits of detection we estimated the sensitivity of the wastewater surveillance platform to detect SARS-CoV-2 transmission as a function of the calculated limits of detection, a correction factor for the population size of the sewershed, and the proportion of houses within the sewershed who are connected to the sewer system using equation 3. The population correction factor in these equations refers to how much larger the sewershed population is than 100,000 population, being one if smaller than 100,000 and otherwise the value of the sewershed population divided by 100,000. We repeated these calculations to estimate the probability that a sewershed had COVID-19 under control. For the probability of control we calculated sensitivity as among all wastewater tests where SARS-CoV-2 RNA was not detected, the proportion of sewersheds reporting < 10 daily incident COVID-19 cases per 100,000 population at the time of the wastewater sample.

(3) \begin{eqnarray*} &&{probability\, of\, absence}\\ &&\quad=\frac{{wastewater\, sensitivity}_{{absence}}}{{population\, correction}}\times{{proportion\, on\, sewer}} \end{eqnarray*}

(4) \begin{eqnarray*} &&{probability\, of\, control}\\ &&\quad=\frac{{wastewater\, sensitivity}_{control}}{population\, correction}\times{proportion\, on\, sewer} \end{eqnarray*}

## Results

### Classification of Wastewater Samples Containing SARS-CoV-2 RNA

We observed that samples could fall into 3 different categories of SARS-CoV-2 detection based on qPCR analysis: below limits of detection, detected but nonquantifiable, or quantifiable. Quantifiable results (33% of samples) could be normalized to crAssphage concentrations to provide a ratio of SARS-CoV-2 to crAssphage, while samples registering below limits of detection (33% of samples) could help support the absence or control of SARS-CoV-2 in an area. Samples containing levels of SARS-CoV-2 RNA lower than the method limits of quantification (34% of samples) were considered nonquantifiable, but still held value because they confirmed SARS-CoV-2 was present in the represented area despite large uncertainties associated with detection at these low levels. Subsequent analysis showed that these classification criteria allowed good agreement between SARS-CoV-2 wastewater and clinical surveillance data.

### Leveraging Wastewater Surveillance Data to Confirm Freedom From Disease

**Fig. 1.**
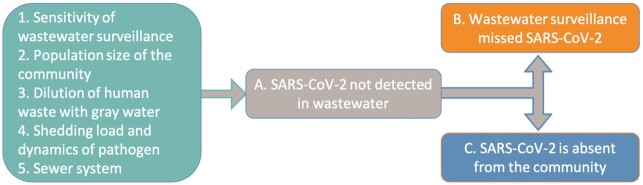
Each time wastewater is tested and SARS-CoV-2 is not detected (**A**), either the system did not detect the SARS-CoV-2 that was in the community (**B**) or there was no SARS-CoV-2 in the community (**C**). The probability of each nondetection representing no SARS-CoV-2 in the community is dependent upon the sensitivity of wastewater surveillance (1), the population size of the community (2), the dilution of human waste with gray water (3), the load shedding dynamics of the pathogen (4), and the sewer system (5). Whereas, each nondetection may only give a low probability that SARS-CoV-2 is absent, repeatedly not detecting SARS-CoV-2 increases the confidence that SARS-CoV-2 is absent using the simple equation of 1-(1-sensitivity)^*n*^ where *n* represents the no. of consecutive nondetections. This process is similar to repeatedly tossing a coin and repeatedly getting heads—each individual coin toss maintains a specific probability but the probability of obtaining a string of repeated consecutive results is increasingly lower with each toss.

**Fig. 2.**
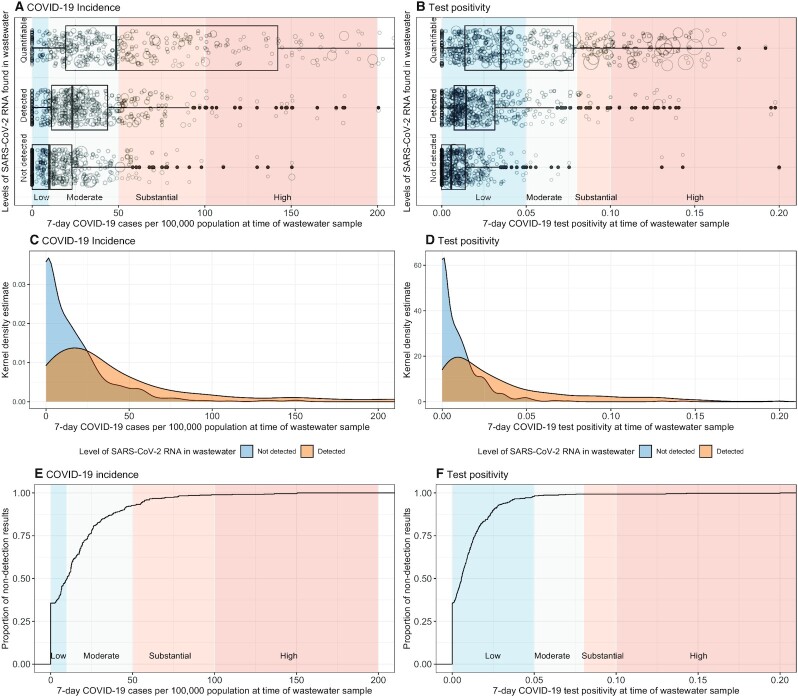
Estimates of the limits of detection (in terms of cases reported in the health system) of SARS-CoV-2 testing in wastewater relative to CDC classification of transmission risk. Clear differentiation in the level of measured community-level COVID-19 incidence (**A**) and test positivity (**B**) when categorizing wastewater results as quantifiable, detected but below the level of quantification, and not detected. Size of the circles in (A) and (B) represent the no. of individuals tested. Large overlap between detection and nondetection exists (**C**) and (**D**), with nondetection of SARS-CoV-2 RNA clustering < 0.5 cases per 10,000 and < 2% test positivity (**E**) and (**F**).

We can leverage negative results from wastewater surveillance to establish the probability that SARS-CoV-2 transmission is below a measurable and manageable threshold using principles of freedom from disease surveillance developed by veterinary scientists (23). Each nondetection of SARS-CoV-2 in wastewater carries1 of 2 possibilities (Fig. 1). Either SARS-CoV-2 is absent from the population or the wastewater test was unable to detect SARS-CoV-2. The ability of wastewater to detect SARS-CoV-2, or the sensitivity of the wastewater surveillance approach in this context, is a function of the method's limits of detection, the population size of the sewershed, dilution of human waste with precipitation and graywater, and the proportion of the population in the sewershed with sewer connections. Using an ultracentrifugation method (22), we observed 35% of 468 wastewater samples with nondetection had 0 weekly reported cases in their respective communities (Fig. 2). Considering the CDC thresholds of < 10 weekly reported COVID-19 cases being at low risk of transmission and 10–50 weekly reported COVID-19 cases being at moderate risk of transmission (24), 50% of wastewater samples with nondetection came from communities with low risk of transmission and 92% of wastewater samples with nondetection came from communities with moderate or lower risk of transmission (Fig. 2). Weekly test positivity was also lower in communities providing a wastewater sample with nondetection, with 65% of wastewater samples having < 1% test positivity in the community and 98% of wastewater samples having < 5% test positivity in the community (CDC's threshold for low transmission). Sewersheds where there were COVID-19 cases reported but no detectable SARS-CoV-2 RNA in wastewater were, on average, 5 times more populous than sewersheds where SARS-CoV-2 RNA went undetected and no COVID-19 case was reported. The no. of diagnostic tests conducted in these more populous sewersheds was slightly higher (a median of 12 compared to 9 per 1,000 population).

By combining negative results (SARS-CoV-2 RNA undetected) over time from wastewater surveillance, we can estimate the probability that a community is free from coronavirus transmission, or at least that transmission is low (less than 10 weekly reported cases per 100,000 population). Importantly, nondetections in larger sewersheds result in less confidence than smaller sewersheds. Among the sewersheds in our study, the potential sensitivity of a single nondetected sample ranged from 0.07 in the largest sewershed (500,000 population) to 0.35 in smaller sewersheds (< 100,000 population) for absence of infection and 0.1–0.5 for low transmission (< 10 weekly cases per 100,000 population). Repeated wastewater samples where SARS-CoV-2 RNA is consistently undetected indicate an increased probability that the upstream community is either free from SARS-CoV-2 transmission or has it under control. This occurred in Cayuga and Cortland counties during the summer of 2020 (Figure S2, Supplementary Material). While individuals in these counties were still at risk of contracting COVID-19 at this time, risk was more dependent on interaction with people outside the community (including recently returned travelers) than interaction with people inside the community. These findings would thus have allowed for social distancing interventions to be more precisely applied.

### Correlation Between Wastewater Results and Pandemic Trajectory

**Fig. 3.**
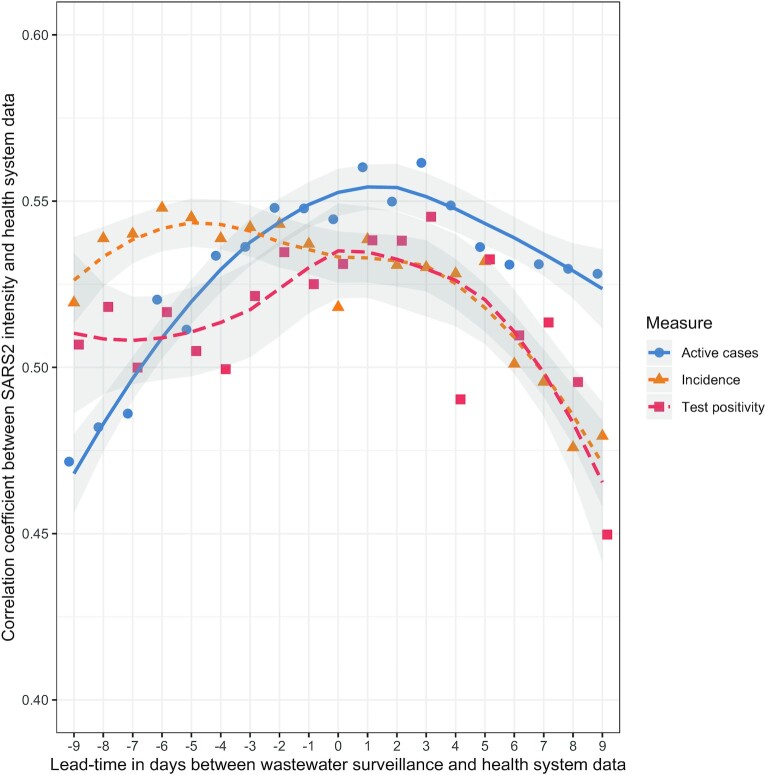
Correlation observed between intensity of SARS-CoV-2 RNA in wastewater and various indicators of SARS-CoV-2 transmission at different lag and lead times, with a locally weighted smoothing (lines) and uncertainty (gray shading).

We observed broad correlation (*⍴*∼0.5) between SARS-CoV-2 intensity in wastewater and COVID-19 case and testing data (Fig. 3; Figure S3, Supplementary Material), including a *⍴* = 0.56 for active cases and *⍴* = 0.55 for a 7-day average test positivity with a 3-day lead between wastewater sample and COVID-19 case data. For a 7-day average of incident COVID-19 cases, we observed a correlation of *⍴* = 0.55 with a 6-day lag between wastewater sample and COVID-19 case data.

### Wastewater Surveillance Provides Early Indication of Increasing Transmission

In the fall of 2020, 3 upstate New York communities monitoring coronavirus in their wastewater reported COVID-19 outbreaks following the return of college students to campus: Cortland, Oneonta, and Oswego. Prior to the beginning of the semester, in late August 2020, SARS-CoV-2 transmission was stable and low with an average incidence between 2020 July 1 and 2020 August 15 of less than 10 weekly reported COVID-19 cases per 100,000 population. Reported COVID-19 cases increased sharply, beginning August 31 in Oneonta and September 10 in both Cortland and Oswego (Figure S4, Supplementary Material). Following a long period of nondetection, SARS-CoV-2 RNA in Cortland wastewater samples was first detected on September 1 and increased to quantifiable levels on September 8 with a continued increase in quantifiable levels thereafter. Following a period of variable detection, but below the level of quantification, SARS-CoV-2 RNA in Oswego wastewater samples was first quantifiable on September 2, with increasing levels thereafter. In Oneonta, quantifiable levels were first observed September 1 and increased thereafter. Together these signals provided about a week's warning of the 3 outbreaks observed (Figure S4, Supplementary Material).

## Discussion

Wastewater surveillance has the capacity to confirm that a community is safe from a pathogen, both through increased confidence in the absence of transmission and the early warning of a pathogen's presence in a community. As a pathogen threatens a community, and particularly when human diagnostics are limited, this benefit is invaluable. These benefits associated with wastewater surveillance are dependent upon methodological limits of detection. In line with reports from Australia (30), we observed an inaccuracy 65% of the time, that being when there is a mismatch between nondetection of SARS-CoV-2 RNA and reported COVID-19 cases in a sewershed. Despite what may appear to be a high rate of inaccuracy, 35% sensitivity in these types of surveillance systems is quite good, as is 50% or 93% sensitivity in a surveillance system to confirm that transmission is low or moderate, respectively. To put these sensitivity estimates in context, in March of 2020 the health system was only about 10% sensitive to COVID-19 cases since as many as 90% of cases of COVID-19 in New York went undiagnosed (31). Additionally, wastewater surveillance is unaffected by health access disparities that lead to underreporting in vulnerable communities (32).

We observed the highest correlation between levels of SARS-CoV-2 RNA in wastewater and health system measures of transmission with a 3-day lead time, similar to Peccia et al (13). Further, we saw greater correlation between active cases and wastewater than we observed with incident cases, suggesting that modelers should take postinfection shedding into account.

Wastewater surveillance will not capture all infections in a community, particularly in very large communities (> 50,000 population) and communities with a high proportion of houses that are not connected to the sewer. (Approximately 80% of NY State's population is connected to the sewer, geographically skewed to urban areas). Fortunately for public health, single infections are less concerning than population-level trends (i.e. spread). With COVID-19 and many other infectious diseases, a majority of infections are dead-end transmission (33, 34). Communities where houses are not connected to sewers would be inherently excluded from a wastewater surveillance program, but we would expect a pathogen to spread between both nonsewered and sewered households. Furthermore, when caseloads are low the test, trace, isolate paradigm should be sufficient for population control (35, 36). Rather than needing to find every instance of a single COVID-19 case in a community, the public and policy makers need to know when a pathogen first arrives in a community and when transmission is increasing beyond chains of known transmission to a state of community spread. In these regards, the sensitivity of wastewater surveillance appears to be more than adequate, at least for the SARS-CoV-2 pathogen.

New York State's health security was breached in 2020 with the undetected arrival and subsequent spread of SARS-CoV-2. The pandemic required broadscale closure of schools, businesses, and general social movement to “flatten the curve” in order to mitigate the surge in hospitalizations and death (37). The only reliable understanding of SARS-CoV-2 transmission at the time of intervention came via hospitalizations that were threatening to overwhelm, and in some cases did overwhelm the health system. Interventions had to be implemented due to increasing case loads and hospitalizations, but by the time these measures increased transmission was already well-established. The infectious disease surveillance system based upon incident cases and hospitalizations missed many cases and could not provide confidence that a community was free from SARS-CoV-2 transmission. Having a wastewater surveillance system in the face of such a public health emergency would have provided a more complete understanding of the geographic extent of SARS-CoV-2 transmission and resulting public health interventions could have been more precise, and perhaps shorter. From our findings, wastewater surveillance as applied in upstate New York exceeds the need in this regard and should be considered an important aspect of a community's health security.

## Supplementary Material

pgac001_Supplementary_Materials

## Data Availability

Anonymized data used in this study are available in the Open Science Framework at https://doi.org/10.17605/OSF.IO/PH36G. All R scripts used in the analyses are available from the authors upon reasonable request.

## Notes

**Competing Interest:** HG and FM hold a provisional patent on the UltraSucrose methodology (application no. 63/039,338) for which Quadrant Biosciences holds an exclusive license.
