# Supplementary material for: Coupling freedom from disease principles and early warning from wastewater surveillance to improve health security
Source: PNAS Nexus. 2022 Mar 2;1(1):pgac001. doi: 10.1093/pnasnexus/pgac001 (PMC9802328; doi:10.1093/pnasnexus/pgac001)
Supplement: pgac001_Supplementary_Materials [file pgac001_supplementary_materials.docx]

**Supplementary Materials:**

Materials and Methods

**Wastewater testing.** Participating wastewater treatment plants, or Arcadis during the New York State pilot, sent a 24-hour composite of 250mL untreated wastewater to the Quadrant Biosciences wastewater analysis laboratory either weekly, twice weekly, or three times weekly depending upon services contracted. Quadrant Biosciences purified and quantified SARS-CoV-2 viral nucleic acid levels contained in wastewater samples by using the ultracentrifugation through sucrose cushion (UltraSucrose) technique followed by qRT-PCR as detailed elsewhere.^22^ Briefly, wastewater was added to a centrifuge tube before adding sucrose solution under the wastewater creating two distinct layers. After ultracentrifugation, the supernatant was removed and the pellet containing nucleic acids was resuspended. Total nucleic acids were extracted from eluted pellets and used immediately as the template for quantification of SARS-CoV-2 and crAssphage DNA and crAssphage RNA. To aid interpretation, SARS-CoV-2 wastewater RNA levels were classified into three distinct categories prior to data analysis. qPCR was run in triplicate. Samples that had all three qPCR replicates amplify above the limit of quantification (LOQ) of 5 genome copies per reaction were classified as quantifiable. Because both assays were able to amplify 5 copies per reaction consistently, samples that had at least one qPCR replicate amplify with a cycle-time threshold (Ct) < 40 were considered detected but not quantifiable (DNQ). Many of the samples classified as DNQ had one or two qPCR replicates above the LOQ of 5 copies but were still conservatively classified as DNQ for our analysis. Samples that had no amplification in any of the three wells (i.e., all three wells were “undetermined”, Ct > 45) were considered below the limits of detection (BLOD), i.e. a “negative” or non-detected sample. The following equation was used to normalize SARS-CoV-2 quantities to the level of fecal material in each sample as indicated by crAssphage DNA concentrations: log_10_(SARS-CoV-2):log10(crAssphage DNA). We imputed a level of 3.5 copies for DNQ samples and 1 copy for BLOD samples prior to log-transformation.

**COVID-19 case data.** COVID-19 case data was pulled from the Electronic clinical Laboratory Reporting System (ECLRS). Every licensed professional authorized by the Department of Health Physician Office Laboratory Evaluation Program to administer a test for COVID-19 or influenza is required to report such results immediately (not more than 3 hours) to the Department of Health through ECLRS when a result is received.^33,34^ COVID-19 cases and tests were retrieved from the New York State ECLRS, addresses were matched with tax parcel data to determine whether the household was connected to public sewer, and then geocoded to sewershed geographies before aggregation into daily numbers. Test positivity was defined as the number of COVID-19 cases divided by the number of COVID-19 tests conducted. The number of active cases was estimated as each COVID-19 case lasting 10 days from diagnosis. We utilized seven-day averages for test positivity and incidence, but not for active cases. We estimated simple Pearson correlations between levels of SARS-CoV-2 RNA in wastewater and measures of incidence, active cases, and test positivity.

**Classification of COVID-19 transmission.** We used the CDC’s guidelines for classifying transmission into low, moderate, substantial, or high depending on the number of weekly cases of COVID-19 per 100,000 population as well as test positivity.^24^ From our results we showed a three-day lead time between wastewater levels and active cases, and so we positioned our week of COVID-19 cases and test positivity accordingly. If incidence and test positivity fall into different risk categories, the CDC recommends taking whichever is higher. For all analyses herein, incidence was higher than test positivity.

**Sewershed population estimates.** We calculated estimates for the 2020 population within each sewershed using R statistical software version 4.0.0.^35^ We first estimated the 2010 population for each sewershed using an overlay of 2010 US census blocks on top of the sewershed boundaries. We calculated the proportion of the area for partial block overlap and then assigned a proportional 2010 decennial population of the block to the sewershed assuming equal distribution of the population in the blocks. We then aggregated the apportioned values to get a total population estimate for the sewershed. We repeated this procedure using 2010 decennial population data for the block group and 2018 American Community Survey (ACS) data for the block group to get 2010 and 2018 population by sewershed based on block groups. We used these values to estimate the rate of population change per sewershed using equation 1. We then applied this average annual change to the sewershed population based on the block data from 2010 and estimated the population after ten years of growth using equation 2 to calculate 2020 population estimates. The “tidycensus” R package provided population estimates^36^ and the “tigris” package provided geometry data.^37^

*Equation 1:* $Annual growth rate = \left( \frac{{2018}_{pop} - {2010}_{pop}}{{2010}_{pop}} \right)/8 years$

*Equation 2:* $2020 population estimate = {2010}_{pop}\times{(1+annual growth rate)}^{10}$

**Freedom from transmission estimates.** In order to estimate the limits of detection of wastewater surveillance we first categorized wastewater results into not detected, detected but below the limits of quantification, and quantifiable. We then examined the reported cases and test positivity in the sewershed as a function of each of these categories. We calculated sensitivity to confirm absence of transmission as among all wastewater tests where SARS-CoV-2 RNA was not detected, the proportion of sewersheds reporting 0 daily incident COVID-19 cases at the time of the wastewater sample. Following the calculated limits of detection we estimated the sensitivity of the wastewater surveillance platform to detect SARS-CoV-2 transmission as a function of the calculated limits of detection, a correction factor for the population size of the sewershed, and the proportion of houses within the sewershed who are connected to the sewer system using equation 3. The population correction factor in these equations refers to how much larger the sewershed population is than 100,000 population, being one if smaller than 100,000 and otherwise the value of the sewershed population divided by 100,000. We repeated these calculations to estimate the probability that a sewershed had COVID-19 under control. For the probability of control we calculated sensitivity as among all wastewater tests where SARS-CoV-2 RNA was not detected, the proportion of sewersheds reporting < 10 daily incident COVID-19 cases per 100,000 population at the time of the wastewater sample.

*Equation 3:* $probability of absence = \frac{{wastewater sensitivity}_{absence}}{population correction}\times proportion on sewer$

*Equation 4:* $probability of control = \frac{{wastewater sensitivity}_{control}}{population correction}\times proportion on sewer$

Supplemental figures:

**

*Supplemental Figure 1: Municipal wastewater treatment plants in New York State. MGD = Million gallons per day.*

*Supplemental figure 2: Probability that coronavirus transmission was absent or under control with repeated non-detection of SARS-CoV-2 RNA in wastewater (open circles) over time alongside incidence of COVID-19 cases and test positivity in the county.*

*Supplemental Figure 3: Trends in SARS-CoV-2 transmission in various communities throughout upstate New York in 2020 as measured by the intensity of SARS-CoV-2 RNA in wastewater, COVID-19 incidence, COVID-19 test positivity, and active COVID-19 cases. Points represent measured SARS-CoV-2 intensity, while lines are smoothed with estimates.*

*
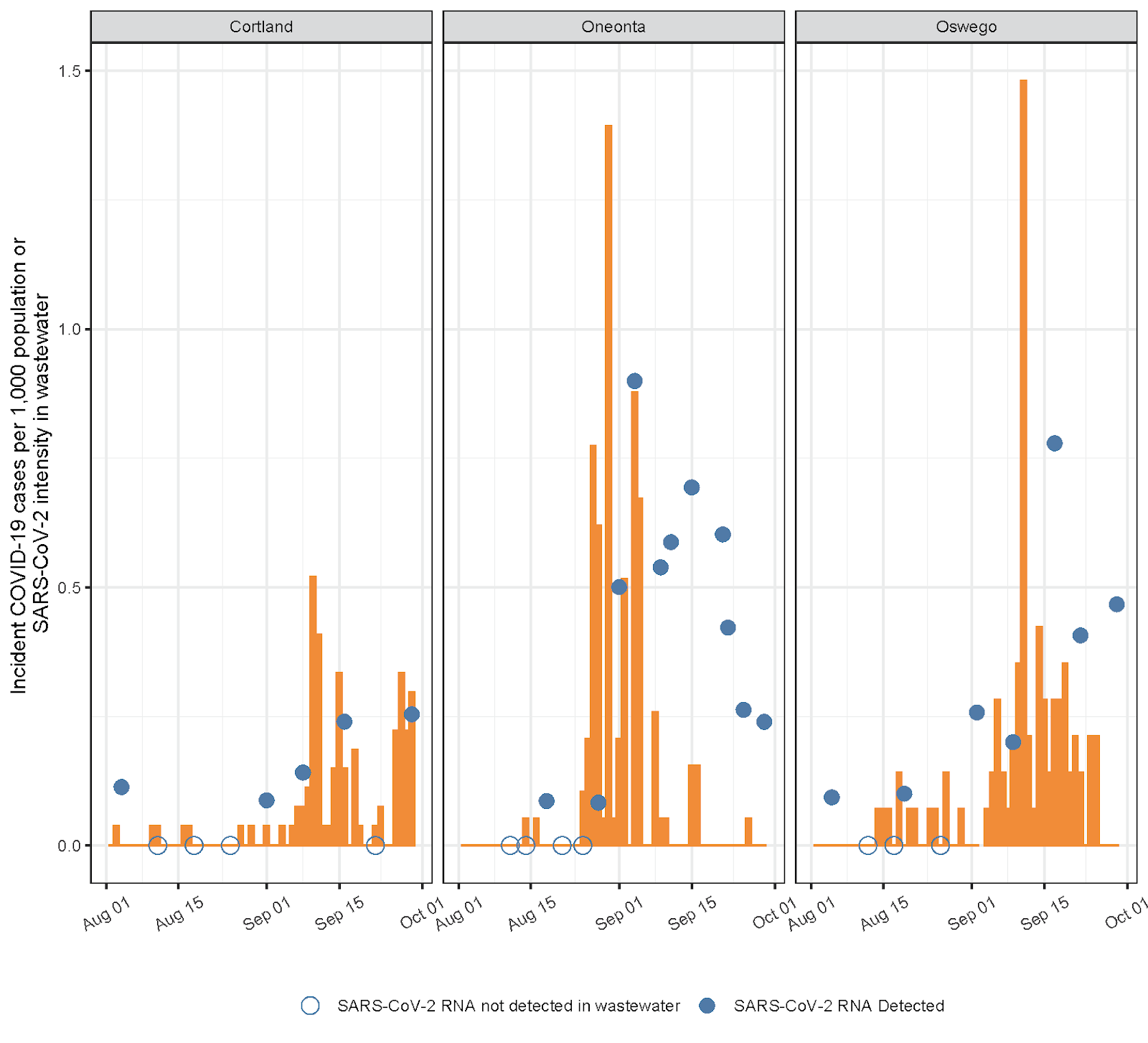
*

*Supplemental figure 4: COVID-19 cases (bars) and wastewater results (circles) from three communities with COVID-19 outbreaks in the fall of 2020. Early indication of increasing transmission was provided by non-quantifiable but detected levels of SARS-CoV-2 RNA in wastewater.*
